# Radiation therapy practice changes in the COVID‐19 pandemic era: A pilot study in California

**DOI:** 10.1002/acm2.13770

**Published:** 2022-08-26

**Authors:** Xiaoyu Liu, Jennifer Zhang, Dan Ruan, Amy S. Yu, Varun Sehgal, X. Sharon Qi, Margaret C. Barker, Zhilei L. Shen, Steve Goetsch

**Affiliations:** ^1^ Department of Radiation Oncology Kaiser Permanente Los Angeles Medical Center Los Angeles California USA; ^2^ Population and Public Health Sciences Keck School of Medicine of University of Southern California Los Angeles California USA; ^3^ Department of Radiation Oncology University of California Los Angeles Los Angeles California USA; ^4^ Department of Radiation Oncology Stanford University Stanford California USA; ^5^ Department of Radiation Oncology University of California Irvine, Orange County California USA; ^6^ Department of Bioengineering University of California Los Angeles Los Angeles California USA; ^7^ Radiation Oncology Ridley‐Tree Cancer Center at Sansum Santa Barbara California USA; ^8^ Department of Radiation Oncology Keck School of Medicine of University of Southern California Los Angeles California USA; ^9^ San Diego Gamma Knife Center Scripps Mercy Hospital San Diego California USA

**Keywords:** COVID‐19, practice changes, radiation oncology

## Abstract

**Purpose:**

This study aims to investigate practice changes among Southern and Northern California's radiation oncology centers during the COVID‐19 pandemic.

**Methods:**

On the online survey platform SurveyMonkey, we designed 10 survey questions to measure changes in various aspects of medical physics practice. The questions covered patient load and travel rules; scopes to work from home; new protocols to reduce corona virus disease‐2019 (COVID‐19) infection risk; availability of telemedicine; and changes in fractionation schedules and/or type of treatment plans. We emailed the survey to radiation oncology centers throughout Northern and Southern California, requesting one completed survey per center. All responses were anonymized, and data were analyzed using both qualitative and quantitative research methods.

**Results:**

At the end of a 4‐month collection period (July 2, 2021 to October 11, 2021), we received a total of 61 responses throughout Southern and Northern California. On average, 4111 patients were treated per day across the 61 centers. New COVID‐19‐related department and hospital policies, along with hybrid workflow changes, infectious control policies, and changes in patient load have been reported. Results also showed changes in treatment methods during the pandemic, such as increased use of telemedicine, hypofractionation for palliative, breast cancer, and prostate cancer cases; and simultaneous boosts, compared to sequential boosts.

**Conclusion:**

Our California radiation oncology center population study shows changes in various aspects of radiation oncology practices during the COVID‐19 pandemic. This study serves as a pilot study to identify possible correlations and new strategies that allow radiation oncology centers to continue providing quality patient care while ensuring the safety of both staff and patients.

## 1 INTRODUCTION

The onset of the COVID‐19 pandemic has dramatically impacted healthcare systems, necessitating quick adoption of new workflows and enhanced safety protocols in order to continue providing quality care to patients while minimizing infection risk to both patients and staff. The field of radiation oncology is no exception to experiencing the challenges brought on by the pandemic. Previous research^1^, ^2^, ^3^ has primarily relied on anecdotal experience of individual radiation center's practices in response to COVID‐19. Previous articles^1^, ^2^ discussed practical suggestions for the radiotherapeutic management of the most frequent cancer during the COVID‐19 era. A joint ESTRO‐ASTRO^1^ strongly recommended stereotactic body radiotherapy (SBRT) for lung cancer treatment. The COVID‐19 pandemic Breast Cancer Consortium^2^ has strongly recommended moderate hypofractionated schemes to reduce treatment duration and patients’ exposure and stated that when an extra dose to the tumor bed is clinically intended, it should preferably be simultaneous and integrated. While previous research articles shared a series of lessons learned and suggestions for other radiation oncology departments to consider when treating cancer patients during the pandemic era, it is desirable to examine comprehensively how different radiation oncology centers have pivoted in response to the pandemic.

To this end, we performed a pilot study to measure the practice changes during the COVID‐19 pandemic among a cohort of 61 of California's radiation oncology centers. This paper discusses changes in patient load experienced during the pandemic, along with adjustments in workflow infrastructure regarding onsite and offsite work. This study reveals the most important changes in infection control policies, work models, and patient treatment methods implemented by the centers to mitigate the unprecedented impact of the COVID‐19 pandemic.

## 2 MATERIALS AND METHODS

The online survey platform SurveyMonkey was used to generate a 10‐question survey, which allowed us to gather data from a large pool of respondents easily and conveniently. The 10 survey questions were designed to assess the impact of the COVID‐19 pandemic on changes in patient load and travel rules; permission and scope to work from home (WFH); new procedures and protocols implemented to reduce COVID‐19 infection risk; availability of telemedicine, and changes in fractionation schedules and/or type of treatment plans. We emailed the survey to medical physicists throughout California, requesting that only one medical physicist per facility complete the survey. Our data collection period spanned 4 months, ranging from July 2, 2021, to October 11, 2021. A total of 61 responses were collected at the end of our collection period. All responses were anonymized, and data were analyzed using both qualitative and quantitative research methods.

## 3 RESULTS

At the end of the 4‐month collection period, we received 36 respondents from Southern California and 25 respondents from Northern California, making a total of 61 responses throughout the state. The survey indicates that on average, 4111 patients were treated per day across the 61 centers in California, with 2461 patients treated from Southern California and 1650 patients treated from Northern California (Table 1).

**TABLE 1:** Number of patients treated per day among Southern and Northern California radiation oncology centers

**Question 1: How many patients do your center treat per day?**		
	**Total number of radiation centers**	**Total number of patients treated/day**
Southern California	36	2461
Northern California	25	1650
Total number	61	4111

Results shown in Table 2 also indicated how these California's radiation oncology centers experienced shifts in patient load due to the pandemic. During our collection period, 52 centers (85.3%) reported that their centers experienced similar patient load as before the pandemic; four (6.5%) centers experienced a decreased patient load as before the pandemic; and five (8.2%) centers experienced an increased patient load, compared to before the pandemic.

**TABLE 2:** Differences in patient load during the pandemic experienced by 61 radiation oncology centers in California

**Question 2: Has your center's patient load remained the same as compared to before the pandemic?**				
	**Yes**	**Decreased**	**Increased**	**Total number**
Southern California	30 (83.4%)	2 (5.6%)	4 (11.2%)	36
Northern California	22 (88.0%)	2 (8.0%)	1 (4.0%)	25
Total number	52 (85.3%)	4 (6.5%)	5 (8.2%)	61

### 3.1 Accommodation of remote working mode

**FIGURE 1:**
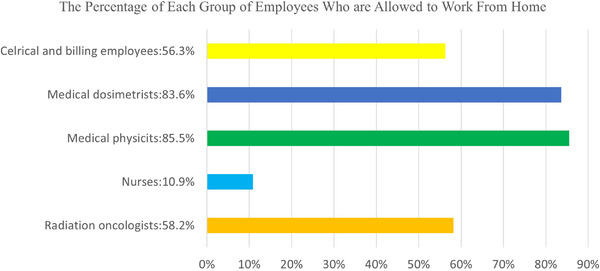
Percentage of different groups of employees who are at least partially allowed to work from home, indicated by 61 radiation oncology centers

Results show close to half of 61 radiation oncology centers suspended travel for employees (52.5%), while the other half did not (47.5%; Table 3). The majority of centers (83.3%) reported using telemedicine during the pandemic, while 16.7% reported not using it. About 90.0% of these centers allowed employees to WFH at least partially. Survey data also show (see Figure 1) the percentages of groups of employees who were allowed to WFH at least partially. Our results show that 56.3% of clerical and billing employees, 83.6% of medical dosimetrists, 85.5% of medical physicists, 10.9% of nurses, and 58.2% of radiation oncologists among our 61 centers were allowed to WFH at least partially.

**TABLE 3:** Measurement of centers that suspended travel for employees and centers that used telemedicine

**Questions**	**Yes**	**No**	**Skipped**	**Total**
Q3: Did your center suspend all travel for employees?	32 (52.5%)	29 (47.5%)	0 (0.0%)	61
Q9: Does your center use telemedicine?	50 (83.3%)	10 (16.7%)	0 (0.0%)	61

### 3.2 Departmental policy changes

Our qualitative data outline the most important department changes indicated by our respondents. We classified practice changes during the COVID‐19 pandemic era into three categories: new COVID‐19‐related department policies, new hybrid work models, and new changes in patient treatment methods. In the category of new COVID‐19‐ related department policies, respondents listed increased infectious control protocols, limiting the number of visitors and required COVID‐19 testing under certain conditions (e.g., identified as close contact and/or experiencing COVID‐19‐like symptoms), as the most important changes within their departments (Table 4). In the category of new hybrid work models, respondents indicated creating staggered onsite work schedules, holding video meetings, and practicing various WFH models, as the most important changes implemented within their departments (Table 4). In the category of new changes in patient treatment methods, respondents indicated that there were increased adoption of hypo‐fractionated of palliative patients, breast cancer, and prostate cancer cases; for example, using 15–16 fractions to treat breast cancer cases, 20–28 fractions to treat prostate cancers; increased SBRT plans, especially for lung cancer and prostate cancer cases; use of hypofractionation simultaneous boosts, compared to sequential boosts; and changes in shifting brachytherapy SAVI to linear accelerator (Linac)‐based treatments as the most important changes implemented within their centers.

**TABLE 4:** Categorization of the most important changes implemented in each radiation oncology center

**Question: What are the most important changes implemented in your center?**		
**New COVID‐19‐related department policies**	**New hybrid work models**	**New changes in patient treatment methods**
Masking requirements	Staggered onsite work schedule	Increased use of hypofractionated of palliative, breast cancer (15–16 frac) and prostate cancer (20–28 frac) patients
COVID‐19 testing required for both patients and staff in certain conditions	Zoom meetings and video conferences	Increased stereotactic body radiotherapy plans for prostate and lung cancer cases
More infectious control protocol: hand washing, temperature check, show proof of vaccination for visitors, and so forth	Hybrid (remote/ in‐person) work from home models	Increased use of hypofractionation and simultaneous boosts, compared to sequential boosts
Limited visitor policy and ancillary/family support to patients		Brachytherapy SAVI moved to Linac‐based treatments
Staff vaccination requirement		

## 4 DISCUSSION

Our California study population averaged a total of 4111 patients treated per day, with each center experiencing different fluctuations in patient load during the pandemic. In order for these centers to continue providing high‐quality radiotherapy to a large volume of cancer patients while keeping employees and patients safe, many centers implemented new infection control policies and changes in patient treatment methods. There are increases in hypofractionation of palliative, breast cancer, and prostate cancer cases and increased use of more hypofractionation and simultaneous integrated boost, compared to sequential boosts.

Our findings provide both qualitative and quantitative insight into how the pandemic has changed practices among 61 of California's radiation oncology centers. We found that many California centers have integrated additional technology and communication platforms to help reduce face‐to‐face interaction and close contact with other staff. In line with the previously reported suggestions by MD Anderson Cancer Center,^3^ our California survey also found that centers had created staggered onsite schedules to reduce onsite capacity while increasing facility‐wide COVID‐19 infectious control policies. Contrary to Knutson et al.’s^4^ departmental practice of moving their entire dosimetry team to remote WFH, our California survey showed that only 83.6% of dosimetrists were allowed to WFH. With 83.3% of radiation oncology centers reporting using telemedicine during the pandemic (Table 3), over 50% of team members were allowed to WFH at least partially (excluding the category of nurses; Figure 1).

Our results may allude to a new direction of integrating WFH into our workflow permanently, and these results provide alternative practice options for radiation oncology centers in the endemic era. Radiation treatment, conventionally considered as one of the personnel‐intensive treatment modalities with the engagement of technical staff, physicists, and physicians, may be amicable to remote working while maintaining high‐quality patient care, with the advancement of technology and communication.

## 5 CONCLUSION

Despite the many disruptions to personal and professional life brought on by the pandemic, COVID‐19 has also created an opportunity for the field of radiation oncology to evaluate our current clinical workflow, safety policies, and patient treatment methods. Although many changes were necessitated by quickly pivoting in response to COVID‐19, these changes may be unanticipated improvements that further modernize our field. It also presented a unique opportunity for medical centers to explore alternative practice options and the feasibility of potentially more effective ways of operation in the endemic era. Our California chapter survey serves as a pilot study to identify possible correlations and change strategies. As the pandemic and its various consequences continue to shape how our radiation oncology centers operate, it is critical to continue investigating to identify, validate, and analyze modifications in response to the pandemic. An extension to a national survey is being planned to verify such changes with the possibility to further reveal demographic‐dependent factors.

## CONFLICT OF INTEREST

The authors declare no conflict of interest.

## AUTHOR CONTRIBUTIONS

Conception and design of the study: Xiaoyu Liu, Jennifer Zhang, Steve Goetsch, Dan Ruan. Acquisition and analysis of the data: Xiaoyu Liu, Jennifer Zhang, Amy S. Yu, Margaret C Barker, X. Sharon Qi, Varun Sehgal, Zhilei Liu Shen, Dan Ruan, Steve Goetsch. Writing and revising the paper: Jennifer Zhang, Dan Run, Xiaoyu Liu, Varun Sehgal, X. Sharon Qi, Amy S. Yu, Zhilei L. Shen. Steve Goetsch. The final manuscript was approved by all these eight authors, and all the authors agreed to be accountable for all aspects of the work in ensuring that the questions related to the accuracy or integrity of any part of the work are appropriately investigated and resolved.

## References

[1] Andrea L , Elisabetta B , Marta B , et al. Radiotherapy in the era of COVID‐19. Expert Rev Anticancer Ther. 2020;20(8):625‐627. doi:10.1080/14737140.2020.1785290 32552073

[2] Dragun AE , Ajkay NJ , Riley EC , et al. First results of a phase 2 trial of once‐weekly hypo fractionated breast irradiation (WHBI) for early‐stage breast cancer. Int J Radiat Oncol Biol Phys. 2017;98:595‐602. 28581400 10.1016/j.ijrobp.2017.01.212

[3] Pollard‐Larkin JM , Briere TM , Kudchadker RJ , et al. Our experience leading a large medical physics practice during the COVID‐19 pandemic. Adv Radiat Oncol.. 2021;6(4):100683. doi:10.1016/j.adro.2021.100683 33824935 PMC8016538

[4] Knutson NC , Kavanaugh JA , Li HH , et al. Radiation oncology physics coverage during the COVID‐19 pandemic: Successes and lessons learned. J Appl Clin Med Phys. 2021;22(3):4‐7. doi:10.1002/acm2.13225 PMC7984470 33742538

